# Analysis of Pulsatile Retinal Movements by Spectral-Domain Low-Coherence Interferometry: Influence of Age and Glaucoma on the Pulse Wave

**DOI:** 10.1371/journal.pone.0054207

**Published:** 2013-01-30

**Authors:** Carolyne Dion, Kanwarpal Singh, Tsuneyuki Ozaki, Mark R. Lesk, Santiago Costantino

**Affiliations:** 1 Institut National de la Recherche Scientifique - Énergie, Matériaux et Télécommunications, Varennes, Québec, Canada; 2 Centre de Recherche de l'Hôpital Maisonneuve-Rosemont, Montréal, Québec, Canada; 3 Department of Ophthalmology, Faculty of Medicine, Université de Montréal, Montréal, Québec, Canada; 4 Biomedical Engineering Institute, Université de Montréal, Montréal, Québec, Canada; Univeristy of Melbourne, Australia

## Abstract

Recent studies have shown that ocular hemodynamics and eye tissue biomechanical properties play an important role in the pathophysiology of glaucoma. Nevertheless, better, non-invasive methods to assess these characteristics *in vivo* are essential for a thorough understanding of degenerative mechanisms. Here, we propose to measure ocular tissue movements induced by cardiac pulsations and study the ocular pulse waveform as an indicator of tissue compliance. Using a novel, low-cost and non-invasive device based on spectral-domain low coherence interferometry (SD-LCI), we demonstrate the potential of this technique to differentiate ocular hemodynamic and biomechanical properties. We measured the axial movement of the retina driven by the pulsatile ocular blood flow in 11 young healthy individuals, 12 older healthy individuals and 15 older treated glaucoma patients using our custom-made SD-OCT apparatus. The cardiac pulse was simultaneously measured through the use of an oximeter to allow comparison. Spectral components up to the second harmonic were obtained and analyzed. For the different cohorts, we computed a few parameters that characterize the three groups of individuals by analyzing the movement of the retinal tissue at two locations, using this simple, low-cost interferometric device. Our pilot study indicates that spectral analysis of the fundus pulsation has potential for the study of ocular biomechanical and vascular properties, as well as for the study of ocular disease.

## Introduction

The pathophysiology of glaucoma, is still under debate: although elevated intraocular pressure (IOP) is the major risk factor [1], there is increasing evidence that the pathogenesis of glaucoma is also linked to the biomechanical properties of the eye [2]–[5] and to abnormal ocular blood flow (OBF) [6], [7]. Both IOP and OBF are pulsatile by nature, being in close relationship with the cardiovascular system activity. [8] Numerous studies have demonstrated reduced IOP pulse amplitudes and reduced pulsatile ocular blood flow in glaucoma patients compared with control subjects[9]–[11]. However, to our knowledge, very little work has been devoted to the analysis of the pulse waveform itself to determine whether it can be used for differentiating diseased from healthy individuals. For instance, Evans *et al*. [12] have shown that the spectral power of the second, third and fourth harmonic components of the IOP pulse is significantly lower in glaucomatous groups compared to normal groups. Unfortunately, assessment of the IOP relies on the use of contact techniques such as pneumotonography [13] or variants of the Goldman’s applanation tonometer [14]. Since Fourier analysis of the pulsatile IOP requires relatively long data acquisition time, which may lead to serious patient discomfort because of direct eye contact, such type of investigation for screening purposes remains unpopular in ophthalmology clinics and researches.

In systemic circulation, however, the use of Fourier analysis to study the pressure and blood flow waveforms has been a standard method of evaluating impedance of the arterial wall. [15], [16] As an example, such analysis can be used to study the influence of vasodilator drugs on the vascular arterial compliance [17] or allow better insights into the vascular status of specific organs, such as in cerebral hemodynamics. [18], [19] In ophthalmology, spectral analysis has been widely used for the study of ocular tremors, [20], [21] pupil size fluctuation, [22], [23] and corneal[24]–[26] and retinal [22], [26], [27] movements, providing better understanding of the ocular deformation in relation with the cardiopulmonary system. More recently, Plumb *et al*. [28] have shown that spectral analysis of the Doppler flow velocity waveform of the ophthalmic artery revealed significant differences in the percentage power of the first four lower frequency sinusoidal components between subjects with type 2 diabetes and healthy controls. Thus, a comprehensive assessment of waveforms can be a very powerful tool for studying pulsatile biomechanical systems.

On the other hand, tremendous efforts have been deployed over the years to elaborate non-invasive instruments to measure pulsatile ocular movements for assessing ocular hemodynamics and/or biomechanics.[4], [22], [24]–[26], [29], [30] We have recently demonstrated the capabilities of spectral-domain low coherence interferometry (SD-LCI) to extract displacement information from sequential axial scans of the eye. [26] This homemade system was successfully used to measure longitudinal retinal and corneal movements *in vivo* in healthy volunteers. [27] It was found that the movements of the cornea and the retina are highly correlated with each other, where the amplitude of both tissues is almost equal. In light of these results, the aim of this work is to demonstrate that the measurement of the longitudinal retinal displacements performed using SD-LCI, along with comprehensive Fourier analysis, can be used to differentiate individuals with different eye hemodynamic and biomechanical properties. Measurements can be achieved with a reliable device, whose principle components consist of a steady low-power broadband laser source, diffractive optics and a camera. We show that the ratio between the fundamental and second harmonic spectral components of the longitudinal retinal movement, the phase shift between these two harmonics, and the phase shift between the movement and the cardiac pulses showed distinctive signatures for the groups varying in age (35 vs 60 years old) and eye condition (normal vs glaucoma).

## Methods

### Experimental Set-up

**Figure 1:**
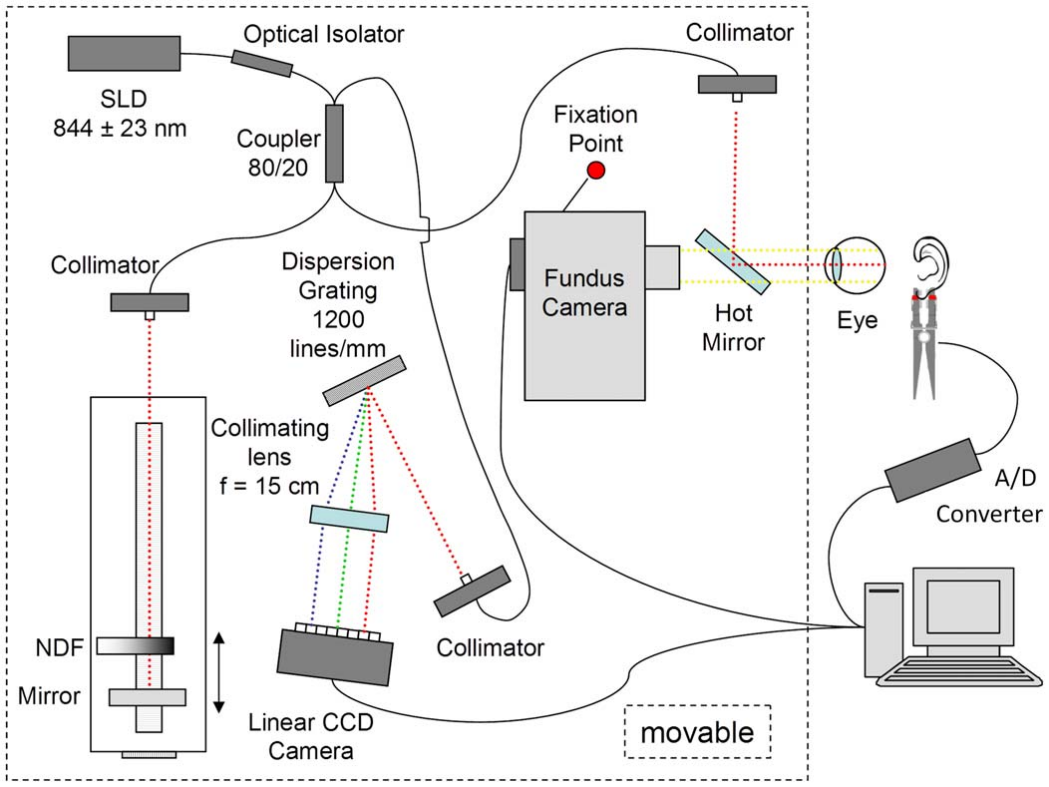
Schematic diagram of a FD-LCI system.

Figure 1 shows a schematic diagram of the optical SD-LCI system. A fiber-based Michelson interferometer was built using a superluminescent diode (SLD, Superlum) with central wavelength of 844 nm and 46 nm bandwidth. The beam was split using a 2×2, 80∶20 fiber coupler into the reference and sample arms terminated with fiber collimators. The collimated sample beam was focused by the eye on to the fundus. The sample arm was also coupled to a fundus camera (Topcon) equipped with an infrared CCD camera (Pulnix), which assured, along with a fixation point, proper positioning of the beam onto the fundus either at the macula or optic disk. The SLD power incident onto the eye was set constant to 700 µW, which is safe for continuous illumination of the human eye according to ANSI safety standards. [31] At the reference arm, the light was reflected by a broadband mirror placed on a motorized translation stage (Zaber Technology). The reference arm beam was attenuated by a neutral density filter (NDF) in order to keep its total intensity below the saturation level of the detector. The two reflected beams (one reflected from the eye layer of interest and the other from the reference mirror) were recombined in the fiber coupler, then dispersed using a diffraction grating (1200 lines/mm) and focused by an achromatic lens (*f = *200 mm) onto a linear CCD camera (Spyder 3, Dalsa Technology). The camera exposure time was set to 400 µs. The full SLD spectrum spanned 700 pixels on the linear CCD camera, which allowed a theoretical depth range of ∼1.2 mm and longitudinal resolution of ∼7 µm in air. The measured sensitivity close to the zero delay position was found to be 94 dB with a decay of 15 dB as one approached 80% of the maximal depth range. Calibration and dispersion compensation of the system were achieved by measuring the position of the peak in the axial scan as a function of the position of the translation stage, repeating this measurement at 100 µm intervals. [32] To establish a relationship between the eye movements and the cardiac pulsations, a custom-made oximeter was placed on the earlobe during measurements to record the pulse of the subjects using a data acquisition card (Labjack, Lakewood, CO). The signals from the linear CCD camera and the oximeter were processed using a dual core 2.0 GHz computer with custom software (LabView, National Instruments). Overall, the total data acquisition time of the CCD camera and oximeter signals provided a sampling rate of 100 Hz. Data was acquired for approximately 20 seconds and stored for later processing.

### Data Analysis

**Figure 2:**
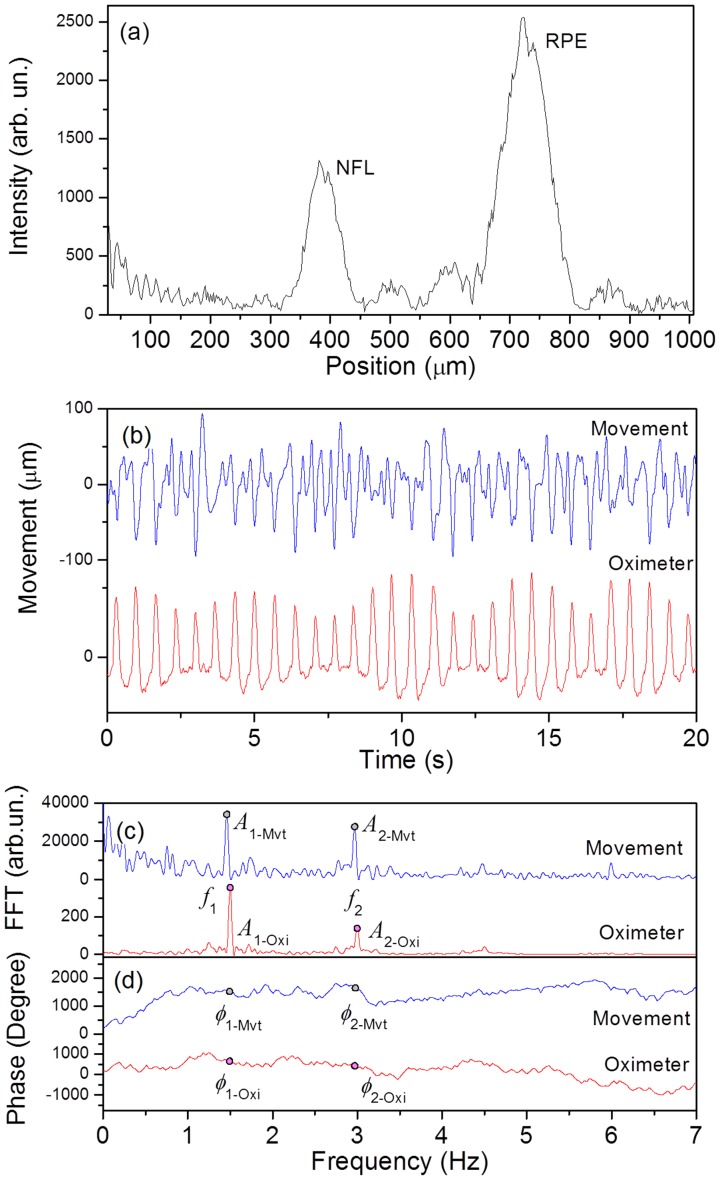
Representative data and movement analysis. (a) Typical A-scan produced by the retina where the nerve fiber layer (NFL) and retinal pigment epithelium (RPE) are clearly observed. (b) Position of the retina in the macula region as a function of time along with oximeter signal, as recorded by the SD-LCI for a 30-years-old normal female subject. Movement and oximeter signal FFT analysis are shown in (c) and (d).

Retinal axial scans (A-scans), or depth profiles, were obtained from the Fast Fourier Transform (FFT) of the SLD spectrum recorded by the CCD camera. A typical A-scan of the retinal layers at the macula region is shown in Fig. 2(a), where FFT peaks can be assigned to reflective interfaces in the sample, which in the present example, to the nerve fiber layer (NFL) and retinal pigment epithelium (RPE). In order to illustrate how the reflectivity profile of the tissues changes during the movements, two A-scans acquired at the retina and the optic disc are plotted simultaneously (Figure S2 and Figure S3 in supplementary information) at extreme time points of the cardiac cycle. For measuring at the optic disc, the probing beam was focused and its position was monitored using a fundus camera throughout the measurement. In order to minimize displacements off the optic disc, the subject was asked to fixate at an illuminated point. As described in a previous paper, [26] measurement of the movement relies on locating the centroid position of FFT peaks in sequential A-scans. An example of such displacement measurements obtained with this procedure is shown along with the oximeter signal in Fig. 2(b). Measurements in which patients blinked for a significant fraction of time were visually identified and discarded for analysis. In addition, frequencies lower than 0.5 Hz and higher than 7 Hz were removed by using a squared band pass filter. No special processing was applied to short blinks, since the frequency filter basically removed their potential contribution. Next, the effective movement amplitude of a given retinal layer was determined by calculating the root mean square of the axial position.

A simple way to inspect the pulse waveform characteristics is to perform Fast Fourier Transform on the pulsatile signal as a function of time. Figure 2(c) and (d) show typical spectral characteristics of the retinal movement and heart signal. The data shown in Fig. 2(c) illustrates that both, the movement and oximeter signals show matched fundamental frequencies (*f*_1_) and second harmonics (*f*_2_). In Fig. 2, for simplicity reasons, data with a narrow spread in the cardiac frequencies is shown, but in several cases the heart signal is non-stationary, which leads to spreading of the frequency components. Such an example is shown in Figure S1.

Although a novel concept in ophthalmology, harmonic components of tissue movements have been found in cardiovascular research to be associated with the vessel’s vascular impedance and wave reflections from the distal arterial tree. [15], [16] From the analysis of spectral power and phase data, additional parameters can also be extracted: (*i*) the ratio between the integrated signal under the second harmonic and fundamental frequency peaks of the movements (*R*_Mvt_ = *A*_2-Mvt/_ *A*_1-Mvt_) and heart signal (*R*_Oxi_ = *A*_2-Oxi/_ *A*_1-Oxi_), (*ii*) the phase shift between the fundamental frequency of the movement with respect to the oximeter fundamental frequency (Θ = Φ_1-Mvt_-Φ_1-Oxi_), and (*iii*) the phase shift between the second harmonic with respect to fundamental frequency for both the movement (Π_Mvt_ = *Φ*_2-Mvt_-*Φ*_1-Mvt_) and the heart signal (Π_Oxi_ = *Φ*_2-Oxi_-*Φ*_1-Oxi_). Integrated signals were computed as the area under each frequency peak in the Fourier space, and phase was also averaged for the same frequency ranges. These three parameters were evaluated and compared for different cohorts of individuals.

### Clinical Subjects

Thirty-eight individuals were recruited from the Maisonneuve-Rosemont Ophthalmology Clinic and Research Center and all experiments were approved by the ethics committee of the Maisonneuve-Rosemont hospital. The procedure and the consequences were described to all the individuals and informed consent was obtained. The procedure adheres to the Declaration of Helsinki. Three cohorts were formed: 11 younger healthy individuals, 12 older healthy individuals, and 15 older treated glaucoma patients. The characteristics of these groups are shown in Table 1. Gender distribution among individuals was similar for the three groups, and age distribution was also similar for the old-healthy and old-glaucoma groups. It is worth mentioning that the three different cohorts were matched for heart rate. Heart rate has been shown to be significantly correlated to measures of the IOP pulse wave, *i.e.* the higher the heart rate, the lower the pulse amplitude, pulse volume and pulsatile ocular blood flow. With the groups matched for heart rate, this factor is eliminated. [12] Diagnosis of glaucoma was made on visual field analysis and optic disk appearance in accordance with established clinical parameters. [33] The average ± standard deviation of the mean defect was found to be 1.61±1.63 dB in glaucoma patients. At the time of study, glaucoma patients had already been treated for their disease with medication controlling increased IOP or laser surgery. The medication used to control the IOP was one of the following; Xalatan die HS, Lumigan HS, Cosopt Bid, Bitoptic Bid, Travatan die HS. For each patient, the eye with greatest optic disk cupping was selected for evaluation. Control healthy subjects had no history of ophthalmic disorders and the results of an ophthalmic examination were normal. The right eye of each individual in both healthy groups was used for evaluation. Neither pupil dilatation nor corneal anesthesia were required for SD-LCI measurements and proper positioning with infrared fundus camera. Axial movements of the retina at both the optic disk and the macula region were measured using the system described above, with participants properly positioned on a chin rest. Several series of twenty-second measurements separated by 1-minute pauses were recorded for each individual. For each patient, data analysis was performed for all series, while data with the best signal-to-noise ratio in the movement FFT spectral power was selected for further comparison. For comparison between different cohorts, data was checked for normality using the Shapiro–Wilk test and results are presented in Table S1. All data sets were found to be normal at a significance level lower than 0.01. Unpaired Student’s *t*-tests were used to compare differences between groups at each eye position. Pearson’s product moment correlation analysis was used to evaluate associations between variables, and *p* values lower than 0.05 were considered statistically significant.

**Table 1:** Characteristics of young healthy, old healthy and old glaucoma subjects.

Parameter	Younger Healthy	Older Healthy	Older Glaucoma
Age (years)	35±7	60±12	60±10
Sex (F/M)	5/6	7/5	9/6
Heart Rate (Hz)	1.19±0.16	1.19±0.12	1.14±0.13
Blood pressure (systolic)	115±10	125±9	118±13
Blood pressure (diastolic)	72±7	81±4	73±7

## Results

**Figure 3:**
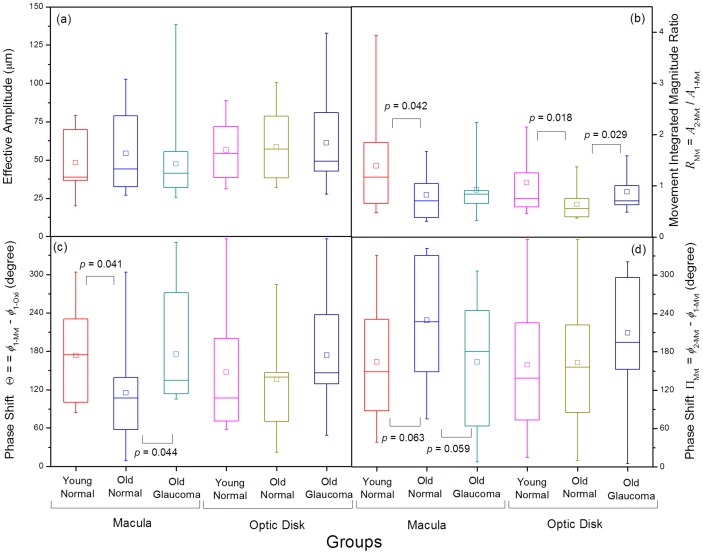
Eye movement at the macula and optic disk region was investigated for the three subject groups. In (a) are compared the effective amplitude of the movement, in (b) the FFT integrated amplitude ratio between the movement fundamental frequency and the movement second harmonic, in (c) the phase shift between the movement fundamental frequency with respect to the oximeter signal fundamental frequency, and in (d) the phase shift between the movement second harmonic with respect to movement fundamental frequency.

Ocular movements at the macula and optic disk regions were analyzed for the three groups. Comparison of the calculated parameters for each of these groups at both the macula and optic disk regions is shown as multiple boxplots in Fig. 3. In Fig. 3(a) the effective amplitude of the movements, which are approximately 50 µm is displayed. Overall, the effective amplitude of the movement seems to be greater at the optic disk than at the macula, but this difference does not reach significance in our small pilot test. In addition, no significant difference in effective amplitude between the three groups is observed. Finally, it should be noted here that this device measures overall changes in the optical path length, this includes not only the fundus pulse amplitude (FPA), but also full-eye displacements. FPA is a few microns (approximately 5 microns), which is one order of magnitude smaller than what we observed as effective amplitude of the retina in our study. This full movement can be measured using a very simple device, but a mechanical interpretation becomes more complicated.

We compared the amplitude of the two most important frequency components. Fig. 3 (b) shows the ratio between the integrated signal under the second harmonic and fundamental peaks of the movements (*R*_Mvt_). Within a specific cohort, there is no significant difference of *R*_Mvt_ between the macula and the optic disk. In most cases, the fundamental frequency integrates the majority of the power as mean *R*_Mvt_ values are below unity. However, younger individual present mean *R*_Mvt_ values higher than one, which means that the second harmonic is the dominant frequency of the movement. Statistical analysis reveals that *R*_Mvt_ is definitely higher in younger healthy individuals than in older healthy subjects at both the macula (*p* = 0.042) and the optic disk (*p* = 0.018). This ratio is also higher for glaucoma patients in comparison to age-matched healthy individuals, though this difference is solely significant at the optic disk (*p* = 0.029).

Next, we compared the temporal delays between the heart signal and the different frequency components. We present in Fig. 3 (c) these phase shifts between the movement fundamental frequency with respect to the oximeter signal fundamental frequency at the macula and the optic disk (Θ). Significant differences between the three groups are obtained at the macula, where both young healthy individuals (*p* = 0.041) and glaucoma patients (*p* = 0.044) present higher phase shift values than the ones obtained for older healthy people.

Finally, we present in Fig. 3 (d) the phase shifts between the second harmonic and the fundamental frequency component of the movement (Π_Mvt_) at the macula and the optic disk. Non negligible differences between the three groups are obtained at the macula, where both young healthy subjects (*p* = 0.063) and glaucoma patients (*p* = 0.059) present lower phase shift values than the ones obtained for older healthy individuals, but these differences did not reach statistical significance.

## Discussion

The results displayed in Fig. 3 showed that the pulsatile eye movement could readily be described in the frequency domain. We found a few parameters that characterize the three groups of individuals by analyzing the movement of the retinal tissue at two locations, using a simple, low-cost interferometric device. Our analysis revealed that the power spectral ratio between the second harmonic and the fundamental frequency components of the movement (*R*_Mvt_), the phase shift between the second harmonic and the fundamental frequency components of the movement (Θ), and the phase shift between the fundamental frequency of the movement with respect to the oximeter fundamental frequency (Π_Mvt_) can be used as parameters to differentiate cohorts with respect to the individuals average age (35 vs 60 years old) and eye condition (normal vs glaucoma). Although, these parameters showed similar tendencies among the various groups, more distinctive differences (lower *p* values) were obtained at the macula. This is probably due to the lower tissue reflectivity at the optic disk compared to the macula, which considerably reduces the overall quality of the data.

An important feature that our study demonstrates is that all groups of subjects showed comparable effective amplitudes of the movement at both the macula and optic disk. This indicates that changes of the eye hemodynamic and biomechanical properties can be detected on the pulse waveform and phase with respect to the heart signal rather than simply the amplitude. The observed differences among different cohorts can be attributed to changes in the biomechanical properties such as stiffening of the eye with age or disease. These biomechanical changes are observed as different time delays between blood-flow-driven oscillations in separate regions of the eye. Nevertheless, it remains speculative to provide an insightful explanation or a mechanical model for this. Although it has been found previously that older eyes are more rigid than the younger eyes, the exact mechanism through which the biomechanical properties of the eye get modified with the progression of the age are not well understood.

Previous investigations of arterial blood pressure have associated higher harmonics in vessel displacement to vascular impedance, which depends on the dimension and viscoelastic properties of arteries, in addition to the viscoelastic properties of the blood. [16] Based on these results, we can postulate that the observed variations of *R*_Mvt_, Θ and Π_Mvt_ are directly related to differences in the ocular vascular status. However, it is important to note that the eye movement from which our analysis is derived is not solely dependent on the eye’s vasculature. Indeed, the retinal tissue kinematics is also likely to dependent on the impedance of scleral tissue. Therefore, even if SD-LCI along with exhaustive spectrum analysis could become a method to characterize ocular tissues, it is still premature to fully identify the hemodynamic status and the biomechanical properties from the eye kinematics.

### Conclusion

Our study showed that spectral decomposition of the pulsatile retinal movements measured with a non-invasive SD-LCI system could be used to characterize the response of retinal and optic nerve head tissues to the cardiac pulsation. The power spectral ratio between the fundamental and second harmonic components of the movement, the phase shift between these two harmonics, and the phase shift between the movement and the cardiac pulses showed distinctive signatures between the groups we compared.

It is important to state that further studies with larger cohorts are required to determine whether our findings will have physiological and clinical utility in glaucoma diagnosis. Furthermore, a better physical description of the movement of the different eyes tissues will help us improve this description and the details of how these movements are described. Overall, this study seeks to show how axial retinal displacements measured with our SD-LCI could be a promising method to differentiate individuals with different ocular hemodynamic and/or biomechanical properties. As a first step towards a potential screening method for glaucoma, we succeeded in defining parameters to group the individuals we analyzed related to their age and eye pathology.

## Supporting Information

Figure S1 **The FFT of recorder movement of the macula (red) and of the heart signal (green) is shown.** The corresponding phase difference between the heart signal and macula movement is shown in blue. The arrows points to the corresponding y-axis.
(DOCX)

Figure S2 **A-Scans of the optic disc for the two extremes of the movement cycle.**
(DOCX)

Figure S3 **A-Scans of the macula for the two extremes of the movement cycle.**
(DOCX)

Table S1 (DOCX)
