# Supplementary material for: Analysis of Pulsatile Retinal Movements by Spectral-Domain Low-Coherence Interferometry: Influence of Age and Glaucoma on the Pulse Wave
Source: PLoS One. 2013 Jan 30;8(1):e54207. doi: 10.1371/journal.pone.0054207 (PMC3559698; doi:10.1371/journal.pone.0054207)
Supplement: Figure S1 — The FFT of recorder movement of the macula (red) and of the heart signal (green) is shown. The corresponding phase difference between the heart signal and macula movement is shown in blue. The arrows points to the corresponding y-axis. (DOCX) [file pone.0054207.s001.docx]

The example provided in Fig. 2 of the main manuscript is a simple case in which the heart signal is composed of only one peak in each harmonics of the FFT signal. However, it is sometimes observed that the heart signal of some of the subjects might is decomposed into multiple frequencies within the same harmonics. One such case is shown in Fig. (A), where the presence of the multiple peaks in the 2^nd^ and 3^rd^ harmonics of the heart signal can be seen. Nevertheless, in such cases, the phase difference between the axial movement and the heart signal for the frequencies contained within the band of a particular harmonics (shown by black solid window) was found to be relatively constant with respect to its surrounding. For example, the standard deviation of the phase difference was found to be 11 and 16 degree for the band of frequencies contained within the 2^nd^ and 3^rd^ harmonics respectively in Fig. (A).





SUPPORTING INFORMATION LEGENDS

Figure S1: The FFT of recorder movement of the macula (red) and of the heart signal (green) is shown. The corresponding phase difference between the heart signal and macula movement is shown in blue. The arrows points to the corresponding y-axis.
