# Supplementary material for: Analysis of Pulsatile Retinal Movements by Spectral-Domain Low-Coherence Interferometry: Influence of Age and Glaucoma on the Pulse Wave
Source: PLoS One. 2013 Jan 30;8(1):e54207. doi: 10.1371/journal.pone.0054207 (PMC3559698; doi:10.1371/journal.pone.0054207)
Supplement: Figure S3 — A-Scans of the macula for the two extremes of the movement cycle. (DOCX) [file pone.0054207.s003.docx]

Figure S3 represents the A-Scans of the macula for the two extremes of the movement, one when the tissue is moving towards the cornea and the other when it moves away. The shift in the position of the A-Scan for the two extremes of the movement can be clearly seen along with a slight change in the reflectivity profile.

SUPPORTING INFORMATION LEGEND

Figure S3: A-Scans of the macula for the two extremes of the movement cycle.
