# Supplementary material for: Analysis of Pulsatile Retinal Movements by Spectral-Domain Low-Coherence Interferometry: Influence of Age and Glaucoma on the Pulse Wave
Source: PLoS One. 2013 Jan 30;8(1):e54207. doi: 10.1371/journal.pone.0054207 (PMC3559698; doi:10.1371/journal.pone.0054207)
Supplement: Table S1 — (DOCX) [file pone.0054207.s004.docx]

|  | Effective Amplitude | | Phase shift  (Π_Mvt_) | | Phase shift  (Θ) | | Movement integrated magnitude ratio (R_Mvt_) | |
| --- | --- | --- | --- | --- | --- | --- | --- | --- |
|  | W | *p* | W | *p* | W | *p* | W | *p* |
| Young Normal (OD) | 0.949 | 0.640 | 0.977 | 0.948 | 0.860 | 0.100 | 0.921 | 0.334 |
| Young Normal (M) | 0.888 | 0.132 | 0.964 | 0.820 | 0.956 | 0.723 | 0.876 | 0.119 |
| Old Normal (OD) | 0.894 | 0.132 | 0.984 | 0.984 | 0.922 | 0.335 | 0.924 | 0.394 |
| Old Normal (M) | 0.894 | 0.132 | 0.893 | 0.130 | 0.947 | 0.605 | 0.936 | 0.450 |
| Old Glaucoma (OD) | 0.908 | 0.150 | 0.924 | 0.200 | 0.949 | 0.483 | 0.890 | 0.100 |
| Old Glaucoma (M) | 0.934 | 0.310 | 0.943 | 0.395 | 0.920 | 0.289 | 0.924 | 0.258 |

Table S1. The table above contains the ‘W’ and ‘*p*’ values of the Shapiro-Wilk normality test performed on sample data sets for optic disc (OD) and macula (M) positions in the eye.
